# A Comparative Study on Analysis of Ginsenosides in American Ginseng Root Residue by HPLC-DAD-ESI-MS and UPLC-HRMS-MS/MS

**DOI:** 10.3390/molecules27103071

**Published:** 2022-05-11

**Authors:** Bo-Yang Hsu, Chen-Te Jen, Baskaran Stephen Inbaraj, Bing-Huei Chen

**Affiliations:** 1 Department of Food and Beverage Management, Lee-Ming Institute of Technology, New Taipei City 243083, Taiwan; byhsu@mail.lit.edu.tw; 2 Department of Food Science, Fu Jen Catholic University, New Taipei City 24205, Taiwan; ricky19960505@gmail.com (C.-T.J.); or 138547@mail.fju.edu.tw (B.S.I.); 3 Department of Nutrition, China Medical University, Taichung 40402, Taiwan

**Keywords:** ginseng root residue, ginsenoside, HPLC-DAD-ESI-MS, UPLC-HRMS-MS/MS, HRMS orbitrap

## Abstract

Ginseng (*Panax quinquefolius*), a popular herbal and nutritional supplement consumed worldwide, has been demonstrated to possess vital biological activities, which can be attributed to the presence of ginsenosides. However, the presence of ginsenosides in ginseng root residue, a by-product obtained during processing of ginseng beverage, remains unexplored. The objectives of this study were to develop a high-performance liquid chromatography-photodiode array detection-mass spectrometry (HPLC-DAD-ESI-MS) and an ultra-high-performance-liquid-chromatography-tandem mass spectrometry (UPLC-HRMS-MS/MS) method for the comparison of ginsenoside analysis in ginseng root residue. Results showed that by employing a Supelco Ascentis Express C18 column (150 × 4.6 mm ID, particle size 2.7 μm) and a gradient mobile phase of deionized water and acetonitrile with a flow rate at 1 mL/min and detection at 205 nm, a total of 10 ginsenosides, including internal standard saikosaponin A, were separated within 18 min and detected by HPLC-DAD-ESI-MS. Whereas with UPLC-HRMS-MS/MS, all the 10 ginsenosides were separated within six minutes by using an Acquity UPLC BEH C18 column (50 × 2.1 mm ID, particle size 1.7 μm, 130 Å) and a gradient mobile phase of ammonium acetate and acetonitrile with column temperature at 50 °C, flow rate at 0.4 mL/min and detection by selected reaction monitoring (SRM) mode. High accuracy and precision was shown, with limit of quantitation (LOQ) ranging from 0.2–1.9 μg/g for HPLC-DAD-ESI-MS and 0.269–6.640 ng/g for UPLC-HRMS-MS/MS. The contents of nine ginsenosides in the ginseng root residue ranged from <LOQ-26.39 mg/g by HPLC-DAD-ESI-MS and <LOQ-21.25 mg/g by UPLC-HRMS-MS/MS, with a total amount of 38.37 and 34.71 mg/g, respectively.

## 1. Introduction

Ginseng, a perennial herb belonging to Araliaceae, has been widely used to promote health due to its anti-tumor [1], anti-inflammation [2] and anti-diabetes [3], as well as neuro and cardiovascular protection [4,5] effects, all of which are believed to be due to presence of the major class of bioactive compounds ginsenosides. Based on their backbone structure, ginsenosides are divided into four types: (1) protopanaxadiol with a dammarane backbone (Rb1, Rb2, Rc, Rd, Rg3 and Rh2); (2) protopanaxatriol with an additional hydroxyl group at C-6 on a dammarane backbone (Re, Rf, Rg1 and Rh1); (3) oleanolic acid with a pentacyclic triterpenoid (Ro) and; (4) ocotillol type with a five-membered epoxy ring at C-20 (Rs) [6,7]. They are further classified as polar ginsenosides (Rb1, Rb2, Rb3, Rc, Rd, Rf, Rg1 and Re) and nonpolar ginsenosides (Rh1, Rh2, Rg2, Rg3, Rg5, Rk1) with white ginseng (dehydrated) possessing only the former type and red ginseng (steamed) possessing both types [7,8]. The variety and amount of ginsenosides in ginseng can be affected by variety, age, part (stem, root, leaf, root hair or berry), growing environment, harvest season and processing method [9,10]. Furthermore, the ginsenoside composition can be used to assess the ginseng variety and such variation in the composition of ginsenosides has been reported to cause significant difference in their biological activity [4,7,11]. Thus, it is important to develop an appropriate analytical method for rapid and sensitive determination of ginsenosides.

Generally, ginsenosides were isolated from ginseng by adopting various extraction techniques including Soxhlet, heat reflux, ultrasonic, solid phase, microwave-assisted, pressurized liquid, enzyme-assisted, accelerated solvent, matrix solid phase dispersion and pulsed electric field [12]. Furthermore, aqueous solutions of methanol or ethanol were commonly used as extraction solvents [9]. Although more than 150 ginsenosides have been identified by various techniques, Rb1, Rb2, Rc, Rd, Re, Rf and Rg1 are the more frequently analyzed ones for quality control of ginseng products [13]. Several analytical methods used for analysis of ginsenosides in ginseng and ginseng products include TLC/HPTLC [14,15], capillary electrophoresis [16], gas chromatography (GC) [17], near infra-red spectroscopy [18], nuclear magnetic resonance (NMR) spectroscopy [19] and HPLC/UPLC [13,20]. The high molecular weight, along with structural and chemical diversity of ginsenosides, have limited the application of several methods. For example, TLC/HPTLC methods show poor separation efficiency and low accuracy, while GC methods are mostly restricted to volatile compounds and usually require a derivatization step. Furthermore, the spectroscopic methods (NIR and NMR) can rapidly analyze ginsenosides with less solvent consumption, but suffer low accuracy in quantification [12].

Among various methods, HPLC methods were the most widely employed analytical technique and their combination with ultraviolet (UV)/photo diode array detection (DAD) or evaporative light scattering detection (ELSD) were the preferred methods for the routine analysis of ginsenosides due to its simplicity and affordability [13,21]. However, poor separation and low sensitivity was shown for some ginsenosides, as well as large solvent consumption, a long analysis time and high baseline noise that can occur for HPLC owing to the weak UV absorption of ginsenosides [12]. Comparatively, mass detectors offer a more sensitive and comprehensive profiling of ginsenosides [10], and high resolution mass spectrometers (HRMS) such as quadrupole time-of-flight (QTOF) and orbitrap enable high resolution of ginsenosides with high mass accuracy [4,22]. Moreover, most UPLC methods employing a short column with small particle size (<2 μm) can not only provide better separation efficiency within a short period of time and higher sensitivity, but also use less solvent to achieve high throughput analysis [22,23].

In this study, an attempt has been made to compare the analysis of 10 ginsenosides including internal standard saikosaponin A in American ginseng root residue, a by-product obtained during processing of ginseng beverage, by HPLC-DAD-ESI-MS and UPLC-HRMS-MS/MS methods. In addition, the effects of mobile phases, column types and sample solvents were compared for the separation efficiency, while both methods were validated for sensitivity, accuracy and precision.

## 2. Results and Discussion

### 2.1. HPLC Analysis of Ginsenoside Standards

**Figure 1:**
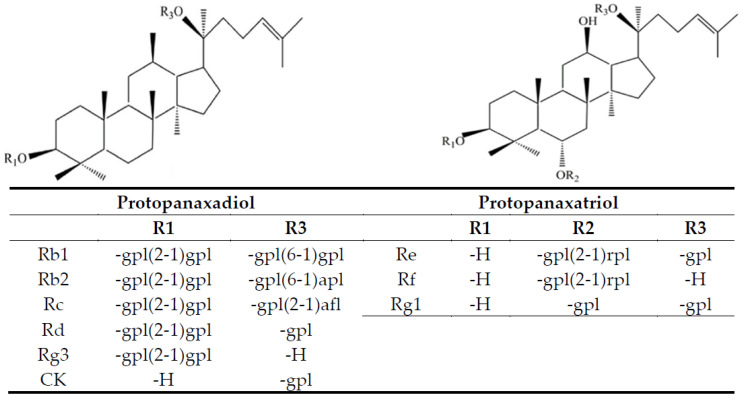
Chemical structures of 9 ginsenosides analyzed in this study. gpl, β-D-glucopyranosyl; apl, α-L-arabinopyranosyl; afl, α-L-arabinofuranosyl; rpl, α-L-rhamnopyranosyl; H, hydrogen.

**Figure 2:**
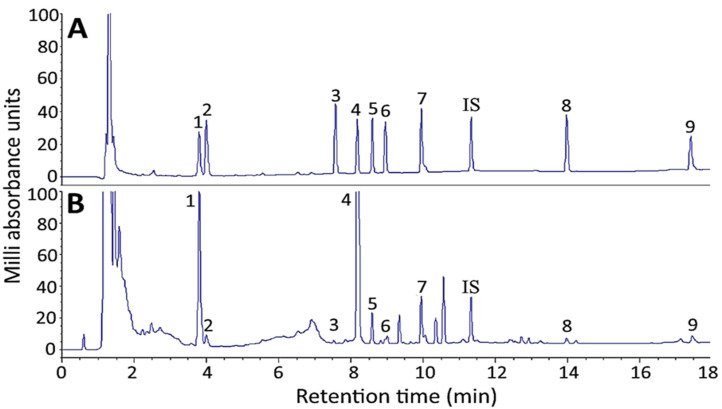
HPLC chromatograms for separation of ginsenosides in standards mixture (**A**) and American ginseng root residue sample (**B**). Separation conditions: column type, Supelco Ascentis Express C18 column; column dimension, 150 × 4.6 mm ID and particle size 2.7 μm; mobile phase, deionized water and acetonitrile; flow rate, 1 mL/min; column temperature, 50 °C; detection wavelength, 205 nm. Peaks: 1, Re; 2, Rg1; 3, Rf; 4, Rb1; 5, Rc; 6, Rb2; 7, Rd; IS (internal standard), saikosaponin A; 8, Rg3; 9, CK.

Figure 1 shows chemical structures of 9 ginsenosides analyzed in this study. Initially a gradient mobile phase of deionized water (A) and acetonitrile (B) used by Cramer and Nold [24] was employed to separate 10 ginsenoside standards including internal standard saikosaponin A. However, the separation efficiency remains inadequate. Thus, by modifying the gradient mobile phase of deionized water (A) and acetonitrile (B) as the following: 75% A and 25% B in the beginning, maintained for 1.5 min, changed to 30% A in 18 min, 0% A in 20 min, a total of 10 ginsenoside standards including Rg1, Re, Rf, Rb1, Rc, Rb2, Rd, Rg3, CK and saikosaponin A were adequately separated within 18 min (Figure 2A). In several previous studies, Chen et al. [25] developed a gradient mobile phase of 0.1% formic acid in water (A) and acetonitrile containing 0.1% formic acid (B) to separate 7 ginsenosides within 30 min by UHPLC-QTOF-MS. Similarly, Uhr et al. [26] developed a gradient mobile phase of 0.02% acetic acid solution (A) and acetonitrile containing 0.02% acetic acid (B) to separate 8 ginsenosides within 70 min by HPLC-MS/MS. In a recent study, Xu et al. [27] developed a gradient mobile phase of acetonitrile (A) and water (B) to separate 19 ginsenosides within 32 min by HPLC. By comparison, the retention time for separation of 10 ginsenosides was much shorter by using the gradient mobile phase in our study and an adequate separation efficiency was attained. Figure 2B shows HPLC chromatogram of ginsenoside in a ginseng root residue sample, and a total of 9 ginsenosides including Re, Rg1, Rf, Rb1, Rc, Rb2, Rd, Rg3 and CK were present with the retention time ranging from 3.79–17.43 min.

### 2.2. Identification and Quantitation of Ginsenosides in Ginseng Root Residue by HPLC-DAD-ESI-MS

Table 1 shows the *m*/*z* value of ginsenoside standards and ginsenosides in ginseng root residue extract by HPLC-MS. A total of 9 ginsenosides including Re, Rg1, Rf, Rb1, Rc, Rb2, Rd, Rg3 and CK were identified in ginseng root residue extract through comparison of *m*/*z* and retention time of ginsenosides in ginseng root residue extract with that of ginsenoside standards (Table 1 and Figure 2). For quantitation, the linear regression equations of Re, Rg1, Rf, Rb1, Rc, Rb2, Rd, Rg3 and CK were prepared with y = 0.6022x + 0.0592, y = 0.7347x + 0.0642, y = 0.7456x + 0.0819, y = 0.5131x + 0.0506, y = 0.5194x + 0.0497, y = 0.4880x + 0.0502, y = 0.6788x + 0.1038, y = 0.8957x + 0.0828 and y = 0.8093x + 0.0184, respectively, and R^2^ all higher than 0.99. Rb1 was found to be present in the highest amount (23,254.89 μg/g), followed by Re (9081.64 μg/g), Rd (1300.39 μg/g), Rc (949.37 μg/g), Rb2 (558.26 μg/g) and Rg1 (305.76 μg/g), while Rf, Rg3 and CK remained undetected (Table 2).

The method validation revealed that the LOD for Re, Rg1, Rf, Rb1, Rc, Rb2, Rd, Rg3 and CK was 0.07, 0.08, 0.20, 0.12, 0.23, 0.21, 0.52, 0.63, and 0.56 μg/g, respectively, while the LOQ was 0.20, 0.23, 0.60, 0.38, 0.69, 0.65, 1.59, 1.90 and 1.69 μg/g.

Table 3 shows the recovery data of ginsenosides by HPLC-DAD-ESI-MS, with the mean recovery being from 101.64% (Rf)-114.32% (Rg3). The repeatability and intermediate precision data are shown in Table 4, with the RSD being from 0.31–2.67% for the former and 1.67–9.18% for the latter. This outcome implied that a high accuracy and precision was attained for the HPLC-DAD-ESI-MS method employed in this study with an insignificant matrix effect for determination of ginsenosides in American ginseng root residue.

### 2.3. Effect of Solvent on Extraction Efficiency of Ginsenosides in Ginseng Root Residue

Table 5 shows ginsenoside contents in ginseng root residue as affected by different extraction solvents based on HPLC-DAD-ESI-MS analysis. Apparently with 80% methanol, 50% ethanol or 80% ethanol as extraction solvent, the highest total ginsenoside content was obtained by HPLC-DAD-ESI-MS analysis, while both 30% and 95% ethanol resulted in a much lower total ginsenoside content. Of the 9 ginsenosides, both Rb1 and Re dominated with Rf, Rb2 and Rg3 remaining undetected. Compared to methanol, ethanol is a much safer solvent and thus 80% ethanol was selected for subsequent extraction and quantitation experiments. In several similar studies Hsu et al. [9] reported a total ginsenoside content of 29.52 ± 6.53 μmol/g in ginseng residue fermented with lingzhi with 80% methanol as extraction solvent at 50 °C for 1 h. Furthermore, a total ginsenoside content of 292.87 ± 1.51 mg/100 g was shown in Korean ginseng with 70% ethanol as extraction solvent at 40 °C for 1 h [28], while that of 45.01 ± 0.1 mg/g was shown in mountain cultivated ginseng with 71% ethanol for extraction 3 times [27]. Comparatively, the total ginsenoside content (36,467.86 μg/g) in ginseng residue obtained by 80% ethanol in our study is comparable to that in ginseng root used for commercial production of ginseng beverage [29]. Furthermore, compared to ginseng root residue, ginseng extract showed a similar individual and total ginsenoside content (data not shown) following the heating of ginseng root sample in 100 °C water for 30 min.

### 2.4. Effect of Sample Solvent on Separation of Ginsenoside Standards

**Figure 3:**
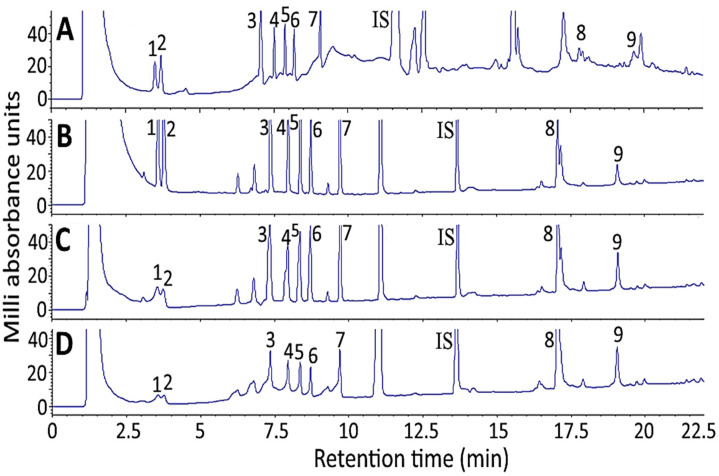
Effect of sample solvent on separation efficiency of 10 ginsenoside standards including internal standard saikosaponin A by HPLC: (**A**) 100% methanol; (**B**) 20% acetonitrile in water; (**C**) 50% acetonitrile in water; (**D**) 80% acetonitrile in water. Separation conditions: column type, Supelco Ascentis Express C18 column; column dimension, 150 × 4.6 mm ID and particle size 2.7 μm; mobile phase, deionized water and acetonitrile; flow rate, 1 mL/min; column temperature, 50 °C; detection wavelength, 205 nm. Peaks: 1, Re; 2, Rg1; 3, Rf; 4, Rb1; 5, Rc; 6, Rb2; 7, Rd; IS (internal standard), saikosaponin A; 8, Rg3; 9, CK.

Figure 3 shows the effect of sample solvent on separation efficiency of 10 ginsenoside standards by HPLC. With 100% methanol as sample solvent, both baseline drift and noise occurred (Figure 3A), probably caused by detection wavelength at 205 nm, which may interfere with the detection of ginsenoside standards as the UV-cutoff wavelength of methanol is also 205 nm. Thus, with a mixture of water and acetonitrile as the sample solvent, the interference was minimized as the UV-cutoff wavelength for water/acetonitrile is 190 nm. Comparatively, with 20% acetonitrile in water as the sample solvent (Figure 3B), the separation efficiency of 10 ginsenoside standards was better than that of 50% acetonitrile in water (Figure 3C) and 80% acetonitrile in water (Figure 3D). Therefore, 20% acetonitrile in water was chosen as the sample solvent for the subsequent experiment.

### 2.5. Effect of Column Type on Separation Efficiency of Ginsenoside Standards

**Figure 4:**
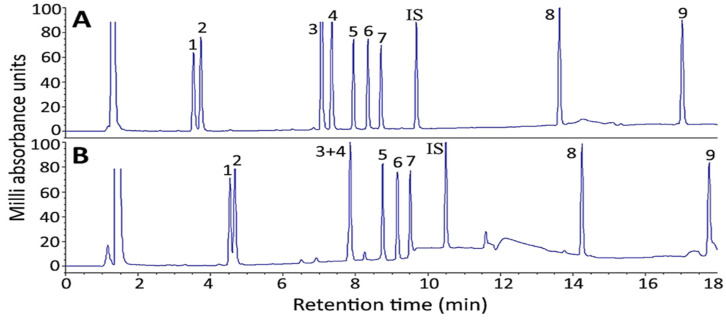
Effect of column type on separation efficiency of 10 ginsenoside standards including internal standard saikosaponin A by HPLC. (**A**), Supelco Ascentis^®^ Express C18 column; (**B**), Waters Cortecs^®^ T3 C18 column. Separation conditions: column dimension, 150 × 4.6 mm ID and particle size 2.7 μm; mobile phase, deionized water and acetonitrile; flow rate, 1 mL/min; column temperature, 50 °C; detection wavelength, 205 nm. Peaks: 1, Re; 2, Rg1; 3, Rf; 4, Rb1; 5, Rc; 6, Rb2; 7, Rd; IS (internal standard), saikosaponin A; 8, Rg3; 9, CK.

Two columns including Supelco Ascentis Express C18 (150 × 4.6 mm ID, particle size 2.7 μm) and Waters Cortecs T3 C18 (150 × 4.6 mm ID, particle size 2.7 μm) were compared with respect to separation efficiency of 10 ginsenoside standards. Apparently a Supelco Ascentis Express C18 column could result in a better separation efficiency of 10 ginsenoside standards, including internal standard saikosaponin A, than a Waters Cortecs T3 C18 column (Figure 4A,B). The difference in separation efficiency between these two columns may be caused by a difference in carbon load, particle size distribution and bonding technique of the C18 stationary phase.

### 2.6. Separation of Ginsenoside Standards by UPLC

**Figure 5:**
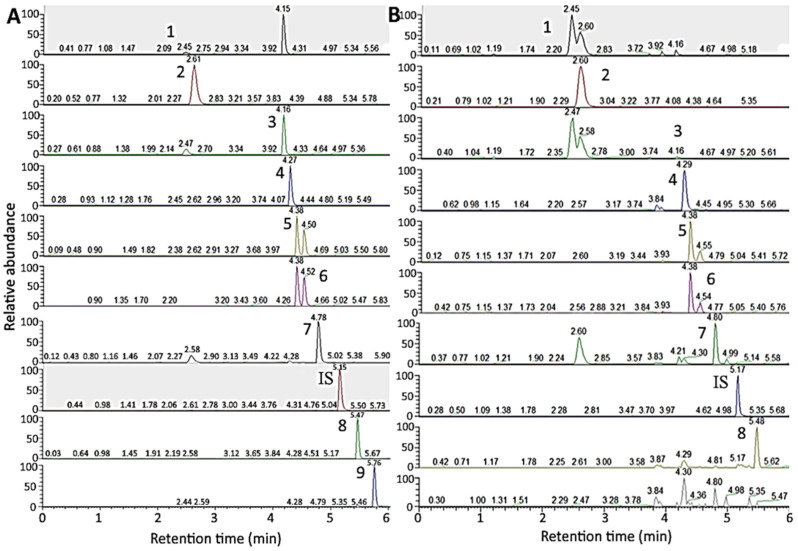
UPLC-HRMS-MS/MS chromatograms for separation and detection of ginsenosides in standards mixture (**A**) and American ginseng root residue sample (**B**) by SRM mode. Separation conditions: column type, Acquity UPLC BEH C18 column; column dimension, 50 × 2.1 mm ID and particle size 1.7 μm; mobile phase, 0.5 mM ammonium acetate and acetonitrile; flow rate, 0.4 mL/min; column temperature, 50 °C; detection mode, selected reaction monitoring (SRM) mode. Peaks: 1, Re; 2, Rg1; 3, Rf; 4, Rb1; 5, Rc; 6, Rb2; 7, Rd; IS (internal standard), saikosaponin A; 8, Rg3; 9, CK.

For UPLC separation of 10 ginsenoside standards including saikosaponin A, an Acquity UPLC BEH C18 column (50 mm × 2.1 mm ID, particle size 1.7 μm, 130 Å) was used with the following gradient mobile phase of 0.5 mM ammonium acetate (A) and acetonitrile (B): 80% A and 20% B in the beginning, maintained for 2 min, changed to 67% A in 3 min, 60% A in 4 min, 50% A in 4.5 min, 5% A in 4.8 min and maintained until 7 min. It was shown that a total of 10 ginsenoside standards including saikosaponin A were adequately separated within 6 min (Figure 5A,B), with flow rate at 0.4 mL/min and detection by SRM mode. In several previous studies Yang et al. [30] used UPLC-MS/MS to separate ginsenosides in *Panax ginseng* by a gradient mobile phase of 0.1% formic acid in water (A) and 0.1% formic acid in acetonitrile (B) to separate 12 ginsenosides within 22 min with flow rate at 0.3 mL/min. Likewise, a total of 26 ginsenosides in *Panax ginseng* were separated within 28 min by using a gradient mobile phase of 0.1% formic acid in water (A) and 0.1% formic acid in acetonitrile (B) with flow rate at 0.5 mL/min by UPLC-QTOF/MS [31]. Furthermore, a total of 15 ginsenosides and 3 aglycones in *Ginseng radix* et rhizome were separated within 36.4 min by using a gradient mobile phase of 0.5 mM ammonium acetate (A) and acetonitrile (B) by UPLC-MS/MS. Compared to HPLC-MS and several previous studies by UPLC-MS/MS, the UPLC mobile phase employed in our study was shown to reduce retention time substantially, maintaining an adequate separation of 10 ginsenoside standards including saikosaponin A.

### 2.7. Identification and Quantitation of Ginsenosides in Ginseng Root Residue by UPLC-HRMS-MS/MS

Table 6 shows retention time, *m*/*z* of precursor ion and product ion as well as collision energy (V) and RF lens (V) of 9 ginsenosides by tandem mass spectrometry (UPLC-HRMS-MS/MS), with retention time ranging from 2.45 min (Rg1) to 5.76 min (CK). The product ions with higher signal intensity were used for quantitation including 638.44 for Rg1 and Re, 475.38 for Rf, 220.98 for Rb1, 945.49 for Rc, 783.39 for Rb2, 783.5 for Rd, 621.43 for Rg3 and 161.05 for CK, while those with lower signal intensity were used for identification including 476.42 for Rg1, 476.54 for Re, 637.44 for Rf, 945.48 for Rb1, 765.47 for Rc, 765.48 for Rb2, 621.44 for Rd, 459.37 for Rg3 and 161.05 for CK. Similar *m*/*z* data used for identification of ginsenosides in ginseng was reported by Uhr et al. [26] and Zhang et al. [32]. Furthermore, the linear regression equations for Re, Rg1, Rf, Rb1, Rc, Rb2, Rd, Rg3 and CK were Y = (2.4670 × 10^−2^) X + (2.3159 × 10^−2^), Y = (8.8520 × 10^−3^) X + (3.7330 × 10^−4^), Y = (10.4032 × 10^−2^) X + (7.8584 × 10^−6^), Y = (4.5967 × 10^−3^) X + (7.5923 × 10^−2^), Y = (9.9804 × 10^−3^) X + (1.5325 × 10^−3^), Y = (6.2406 × 10^−3^) X + (2.3401 × 10^−4^), Y = (2.1853 × 10^−2^) X + (1.0420 × 10^−2^), Y = (3.3966 × 10^−2^) X + (5.8098 × 10^−4^) and Y = (7.2961 × 10^−3^) X + (2.3964 × 10^−5^), respectively, and R^2^ being all higher than 0.99.

The method validation revealed that the LOD of Re, Rg1, Rf, Rb1, Rc, Rb2, Rd, Rg3 and CK was 0.089, 2.133, 0.705, 1.373, 1.553, 2.191, 1.039, 0.912 and 0.089 ng/g, respectively, while the LOQ was 0.269, 6.463, 2.135, 4.161, 4.707, 6.640, 3.147, 2.763 and 0.269 ng/g, respectively. The recovery data of ginsenosides by UPLC-HRMS-MS/MS is shown in Table 7 and the mean recovery ranged from 86.82% (Rf) to 100.83% (Rc), implying an insignificant matrix effect for determination of ginsenosides in American ginseng root residue. Table 8 shows the repeatability and intermediate precision data of ginsenosides with the RSD being from 1.44–7.08% for the former and 3.76–8.31% for the latter. Taken together, all the recovery and precision data of HPLC-DAD-ESI-MS and UPLC-HRMS-MS/MS met the method validation guideline issued by Taiwan Food and Drug Administration (TFDA) [33], implying that the method developed in our study for determination of ginsenosides in ginseng root residue possessed high accuracy and precision.

Following quantitation by UPLC-HRMS-MS/MS, Rb1 was present in the largest amount (21,256.18 μg/g), followed by Re (7958.80 μg/g), Rd (1935.39 μg/g), Rc (1262.94 μg/g), Rg1 (934.56 μg/g), Rb2 (583.68 μg/g), Rg3 (353.16 μg/g), Rf (4.71 μg/g) and CK (<LOD) (Table 2).

It has been well documented that ginsenoside contents can be varied depending on part and variety of ginseng. For instance, Kang and Kim [28] compared ginsenoside contents in different parts of Korean ginseng and reported that the total ginsenoside content was present in the highest amount in ginseng leaf (3538.71 mg/100 g), followed by ginseng root hair (1186 mg/100 g) and ginseng root (292.87 mg/100 g). More specifically, Rh1, Rb3 and Rd were present at a high level in ginseng leaf, while both Rc and Re were present at a high level in ginseng root hair and Rc, Rb1, Rg1 and Rh1 at a high level in ginseng root [28]. Similarly, Li et al. [34] compared ginsenoside contents in *Panax ginseng* and *Panax quinquefolius* leaves, with Re showing the highest level (7.394–8.286 mg/g), followed by F1 (4.409–4.770 mg/g), Rd (3.390–3.737 mg/g), Rb2 (2.635–2.792 mg/g), Rg1 (2.405–3.996 mg/g) and Rc (1.098–1.211 mg/g) for the former, while for the latter, Re was present at the highest level (8.162 mg/g), followed by Rb3 (4.977 mg/g), Rb2 (3.543 mg/g), Rd (3.424 mg/g), F1 (3.271 mg/g) and Rg1 (1.644 mg/g). Comparatively, in our study both Rb1 and Re dominated in ginseng root residue prepared from ginseng root with a much higher level than that reported in the literature.

Compared to HPLC-DAD-ESI-MS, the total ginsenoside content in ginseng root residue as determined by UPLC-HRMS-MS/MS was similar (Table 2). However, three ginsenosides including Rf, Rg3 and CK remained undetected in ginseng root residue by HPLC-DAD-ESI-MS, while only CK was undetected by UPLC-HRMS-MS/MS. Moreover, it is evident that the contents of Re and Rb1 were higher in American ginseng root residue by HPLC-DAD-ESI-MS, while Rg1, Rc, Rb2 and Rd were higher by UPLC-HRMS-MS/MS, which may be attributed to the difference in the analytical technique (HPLC versus UPLC), separation conditions and detector signal response (single quadrupole ESI-MS versus HRMS-MS/MS) for each ginsenoside. This outcome implied that UPLC-HRMS-MS/MS possessed a much higher sensitivity than HPLC-DAD-ESI-MS, as evident by a much lower LOD and LOQ of 9 ginsenosides when detected by the former.

Obviously, the orbitrap HRMS tandem spectrometry detection provides high resolution and mass accuracy with improved scan rates to attain 60,000–100,000 resolution at 1 Hz for generating adequate signal intensity even for an UPLC peak width of 5–20 s [35]. Compared to triple-quadrupole mass detection, HRMS can provide higher sensitivity by eliminating background noise as well as facilitating higher selectivity and specificity by differentiating compounds with small mass difference. Furthermore, unlike triple-quadrupole, HRMS mass detection by orbitrap or QTOF enables both qualitative and quantitative analyses of samples with multiple targets and compounds involving complex fragmentation patterns [35]. HRMS also allows faster method development with minimal compound optimization.

Among the 9 ginsenosides determined in this study, the ginsenoside Rb1 was shown to be present in the largest amount in American ginseng root residue. Generally, Rb1 is abundant in roots, rhizomes and root hairs of ginseng when compared with stem and leaves. Numerous reports have shown that Rb1 is responsible for most of the ginseng plant’s pharmacological activity especially in the cardiovascular, endocrine and immune systems [36]. Most importantly, Rb1 is the key component for neuroprotection with a recent finding suggesting a greater neuroprotective activity for Rb1 when administered intranasally [37]. A recent review on analysis of preclinical evidence of Rb1 highlighted that its neuroprotective effect was mainly through attenuating brain water content, promoting the bioactivity of neurogenesis, antioxidative, anti-inflammatory and anti-apoptosis effects with enhanced cerebral circulation and energy supplementation [38]. In a study dealing with anti-amnestic and anti-aging effects, Cheng et al. [39] demonstrated the potential of Rb1 and Rg1 to attenuate the neurogenerative disorders by increasing neural plasticity and proliferation/differentiation of neural progenitor cells in dentate gyrus of hippocampus of normal adult mice and global ischemia model in gerbils. In addition, Yang et al. [40] reported the cardioprotective effects of Rb1 through protection of cardiomyocytes from oxygen-glucose deprivation injuries by targeting microRNA-21 and its target gene programmed cell death protein 4 (PDCD4) in oxygen-glucose deprivation (OGD)-injured cardiomyocytes. Several recent studies have also reported the antidiabetic, anti-obesity and anti-aging effects as well as osteogenesis and angiogenesis effects to alleviate bone disorders. For instance, Zhou et al. [41] have shown that Rb1 could exert protective effect on diabetes by regulating mitochondrial energy metabolism, improving insulin resistance and alleviating the diabetic complications, while Guo et al. [42] demonstrated its anti-obesity activity through the reduction in body weight and improvement in glycolipid metabolism by upregulating proliferator activated receptor gamma (PPARγ) and aquaporin 7 (AQP7) protein levels. More recently, Wu et al. [43] showed that Rb1 could reinforce the osteogenesis differentiation and angiogenesis factor’s expression of bone mesenchymal stem cells. 

## 3. Materials and Methods

### 3.1. Materials

American ginseng (*Panax quinquefolius*) root was procured from a local shop in Taipei, Taiwan, and placed into several plastic bags for vacuum sealing and storage at −30 °C for use.

Ginsenoside standards including Rb1, Rb2, Rc, Rd, Re, Rf, Rg1, Rg3, CK and internal standard saikosaponin A were purchased from Advanced Chemical Co. (Taichung, Taiwan). HPLC-grade solvents including methanol and acetonitrile were from Merck Co. (Darmstadt, Germany), while ethanol (99%) and dimethyl sulfoxide (DMSO) were from Sigma-Aldrich Co. (St. Louis, MO, USA). Deionized water was made using a Milli-Q water purification system from Millipore Co. (Bedford, MA, USA). Three columns including Acquity UPLC BEH C18 column (130 Å, particle size 1.7 μm, 50 × 2.1 mm ID), Supelco Ascentis Express C18 (150 × 4.6 mm ID, particle size 2.7 μm) and Waters Cortecs T3 C18 (150 × 4.6 mm ID, particle size 2.7 μm) were obtained from Yu-Ho Co. (New Taipei City, Taiwan). 

### 3.2. HPLC and UPLC Instruments

The HPLC-DAD system (1200 series), composed of an on-line degasser (G1379B), quaternary pump (G1312B BIN pump), autosampler (1260 Infinity G1329B 1260 ALS), column temperature controller (G1316B TCC SL) and photodiode array detector (DAD, G1315C DADSL), was from Agilent Technologies Co. (Santa Clara, CA, USA). The single quadrupole mass spectrometer (6130) with multi-mode ion source (ESI and APCI) was also from Agilent Technologies Co. The UPLC system (Accela 600 series) with LTQ Orbitrap XL of a tandem mass spectrometer (UPLC-HRMS-MS/MS) was from Thermo Fisher Scientific Co. (San Jose, CA, USA).

### 3.3. Effect of Solvent on Extraction Efficiency of Ginsenoside from Ginseng Root Residue

A method based on Hsu et al. [9] was modified to extract ginsenoside from ginseng root residue. Initially a 100-g ginseng root sample was mixed with 1000 mL of deionized water in a flask and heated at 100 °C for 30 min, after which the ginseng root residue was collected, freeze-dried and ground into powder for subsequent extraction. Then one gram of dried sample was mixed with 30 mL of various solvents including 80% methanol, 30% ethanol, 50% ethanol, 80% ethanol and 95% ethanol separately for comparison of extraction efficiency, after which each solution was shaken in a 50 °C water bath (150 rpm) for extraction for two hours, followed by filtering through a Whatman No. 2 filter paper (Advantec Co., Tokyo, Japan). The residue was then repeatedly extracted once with the same solvent and both filtrates were combined, evaporated to dryness at 40 °C, dissolved in 10-mL of 20% acetonitrile, filtered through a 0.22-μm membrane filter, and 20 μL injected into HPLC-DAD-ESI-MS, while 5 μL injected into UPLC-HRMS-MS/MS.

### 3.4. Analysis of Ginsenosides in Ginseng Root Residue by HPLC-DAD-ESI-MS

A method based on Cramer and Nold [24] was modified to separate ginsenosides in ginseng root residue. Two columns including Supelco Ascentis Express C18 and Waters Cortecs T3C18 as mentioned in the materials section were compared with respect to the separation efficiency of 10 ginsenoside standards including internal standard saikosaponin A with a gradient mobile phase of deionized water (A) and acetonitrile (B): 75% A and 25% B initially, maintained for 1.5 min, decreased to 15% A in 12 min, maintained for one minute and returned to 75% A in 15 min. The flow rate was one milliliter per minute with detection wavelength at 205 nm and column temperature at 50 °C. For identification by HPLC-MS, a single quadrupole mass spectrometer with electrospray ionization (ESI) in negative mode was used in the scanning range of *m*/*z* 100–1000, drying gas flow 10 L/min, nebulizer pressure 35 psi, drying temperature 350 °C, vaporizer temperature 250 °C, capillary voltage 4000 V (positive and negative), corona current 4 μA (positive) and 40 μA (negative), charging voltage 2000 V (positive and negative), and fragmentor voltage 200 V.

For quantitation, a total of nine concentrations (0.5, 1, 2.5, 5, 10, 25, 50, 100 and 200 μg/mL) were prepared separately for all the nine ginsenoside standards including Re, Rg1, Rf, Rb1, Rc, Rb2, Rd, Rg3 and CK. Then the internal standard (saikosaponin A) was mixed with each standard for a final concentration at 10 μg/mL. Following injection into HPLC-DAD-ESI-MS, the standard calibration curves of nine ginsenosides were prepared separately by plotting the concentrations ratio (Cs/Ci) against the peak area ratio (As/Ai) for the calculation of linear regression equations and coefficient of determination (R^2^).

### 3.5. Method Validation

Based on a standard protocol by International Conference on Harmonization (ICH) [44], both limit of detection (LOD) and limit of quantitation (LOQ) were determined. Briefly, three concentrations (0.2, 0.6 and 1 μg/mL) were prepared separately for Re, Rg1, Rb1, Rc and Rb2 standards containing internal standard at 10 μg/mL, while three concentrations (1, 3, and 10 μg/mL) prepared separately for Rf, Rd, Rg3 and CK standards containing the same internal standard. Following triplicate injections, the standard curves were obtained by a plotting concentration ratio (Cs/Ci) against the peak area ratio (As/Ai), and the slope (s), intercept (I) and standard deviation of intercept (SD_I_) were obtained for determination of LOD and LOQ using the following formula:

(1) $$\mathrm{LOD}=3.3\;\times \;\left(\frac{{\mathrm{SD}}_{I}}{{\mathrm{Mean}}_{s}}\right)$$

(2) $$\mathrm{LOQ}=10\;\times \;\left(\frac{{\mathrm{SD}}_{I}}{{\mathrm{Mean}}_{s}}\right)$$

where:

Mean_s_: mean value of slope.
SD_I_: standard deviation of intercept.

For recovery determination, two concentrations of ginsenoside standards including Rg1 (200 and 400 μg/g), Re (1000 and 2000 μg/g), Rf (60 and 120 μg/g), Rb1 (2500 and 5000 μg/g), Rc (500 and 1000 μg/g), Rb2 (150 and 300 μg/g), Rd (250 and 500 μg/g) and Rg3 (190 and 380 μg/g) were added separately to a ginseng root residue sample for extraction and HPLC-DAD-ESI-MS analysis in five replicates. After quantitation, the recovery of each standard was calculated based on the relative ratio of the standard amount after HPLC analysis to the standard amount spiked before HPLC analysis using the following formula:

(3) $$\mathrm{Recovery}=\left(\frac{\mathrm{amount}\;\mathrm{found}-\mathrm{original}\;\mathrm{amount}}{\mathrm{amount}\;\mathrm{spiked}}\right)\times 100$$

For precision study, the repeatability was carried out by analyzing ginsenoside contents in ginseng root residue samples in five replicates on the same day, while the intermediate precision was performed by analyzing ginsenoside contents in ginseng root residual samples in five replicates one day for three consecutive days. Then the coefficient of variation (RSD, %) was obtained for both repeatability and intermediate precision.

### 3.6. Analysis of Ginsenosides in Ginseng Root Residue by UPLC-HRMS-MS/MS

A method based on Zhang et al. [32] was modified to separate 10 ginsenoside standards as mentioned above by using an Acquity UPLC BEH C18 column (50 mm × 2.1 mm ID, 1.7 μm, 130 Å) with a gradient mobile phase of 0.5 mM ammonium acetate (A) and acetonitrile (B): 80% A and 20% B initially, maintained for two minutes, decreased to 67% A in three minutes, 60% A in four minutes, 50% A in 4.5 min, 5% A in 4.8 min, maintained until seven minutes and returned to 80% A in eight minutes. The flow rate was 0.4 mL/min with column temperature at 50 °C and detection by selected reaction monitoring (SRM) mode. The MS conditions were as follows: negative ion spray voltage at 3000 V, sheath gas flow rate at 38 arbitrary units, auxiliary gas flow rate at 12 arbitrary units, sweep gas flow rate at zero arbitrary units, ion transfer tube temperature at 350 °C and vaporizer temperature at 250 °C. 

For quantitation, a total of 17 concentrations (0.1, 0.2, 0.4, 0.6, 0.8, one, two, four, six, eight, 10, 20, 40, 60, 80, 100 and 200 μg/mL) were prepared and seven concentrations were selected separately for a specific ginsenoside standard, followed by adding saikosaponin A to each standard for a final concentration at 10 μg/mL. Following triplicate injections of each standard to UPLC-HRMS-MS/MS, all the standard curves were obtained separately by plotting concentration ratio (Cs/Ci) against peak area ratio (As/Ai), with the linear regression equations and coefficient of determination (R^2^) being calculated.

### 3.7. Method Validation

Following the same approach for determination of LOD and LOQ by HPLC-DAD-ESI-MS as shown above, three concentrations (two, four and six micrograms per milliliter) of Re, Rb2, Rg3 and CK containing saikosaponin A at 10 μg/mL were prepared separately, while three concentrations (one, two and four micrograms per milliliter) of Rb1, Rc and Rd as well as three concentrations (0.5, one and two micrograms per milliliter) of Rg1 and Rf containing the same internal standard at 10 μg/mL were also prepared. Following triplicate injections of each standard concentration, the slope (s), intercept (δ) and standard deviation of intercept (SD_δ_) were obtained from standard curves for LOD and LOQ determination using a formula shown above. 

For recovery determination, two concentrations of ginsenoside standards including Rg1 (450 and 900 μg/g), Re (1000 and 2000 μg/g), Rf (2.5 and 5 μg/g), Rb1 (2500 and 5000 μg/g), Rc (500 and 1000 μg/g), Rb2 (250 and 500 μg/g), Rd (200 and 400 μg/g) and Rg3 (100 and 200 μg/g) were separately added to a ginseng root residue sample for extraction and UPLC-HRMS-MS/MS analysis in five replicates. After quantitation, the recovery of each standard was calculated using the same method shown above. For precision study, both repeatability and intermediate precision was also conducted by using the same procedure shown above.

### 3.8. Statistical Analysis

All the data were subjected to statistical analysis by using a statistical analysis system (SAS) software [45]. Furthermore, the analysis of variance was conducted by ANOVA and Duncan’s multiple range test for significance in mean comparison (*p* < 0.05).

## 4. Conclusions

In conclusion, both HPLC-DAD-ESI-MS and UPLC-HRMS-MS/MS methods were compared with respect to separation efficiency of nine ginsenosides in ginseng root residue plus internal standard saikosaponin A, with the retention time being 18 min for the former and six minutes for the latter. Both methods showed high accuracy and precision with the total ginsenoside contents being similar. However, the UPLC-HRMS-MS/MS method showed a much higher sensitivity than the HPLC-DAD-ESI-MS method, as evident from the low LOD and LOQ values obtained for the former method.

## Figures and Tables

**Table 1:** The *m*/*z* value of ginsenoside standards and ginsenosides in American ginseng root residue extract by HPLC-MS ^a^.

Ginsenosides	Retention Time (min)	*m*/*z* Value ^b,c^	
		Ginsenoside Standard	American Ginseng Root Residue
Re	3.79	945.5 [M-H]^−^, 981.4	945.5 [M-H]^−^, 981.5
Rg1	4.03	799.5 [M-H]^−^, 835.4	799.4 [M-H]^−^, 835.4
Rf	7.59	799.4 [M-H]^−^, 835.4	800.1 [M-H]^−^, 835.4
Rb1	8.21	1107.5 [M-H]^−^, 1143.5	1107.5 [M-H]^−^, 1143.5
Rc	8.63	1077.5 [M-H]^−^, 1113.5	1077.5 [M-H]^−^, 1113.5
Rb2	8.95	1077.5 [M-H]^−^, 1113.5	1077.5 [M-H]^−^, 1113.5
Rd	9.98	945.5 [M-H]^−^, 981.5	945.5 [M-H]^−^, 981.5
Rg3	13.98	783.4 [M-H]^−^, 819.4	783.5 [M-H]^−^, 819.4
CK	17.43	621.4 [M-H]^−^, 657.4	621.4 [M-H]^−^, 657.4

^a^ The mass spectral identification data by HPLC-MS are based on a method described in Section 3.4. ^b^ *m*/*z*, mass-to-charge ratio. ^c^ two *m*/*z* values for each ginsenoside represent [M-H]^−^ and [M-H]^−^ + 2H_2_O values.

**Table 2:** Contents of ginsenosides in American ginseng root residue by HPLC-DAD-ESI-MS and UPLC-HRMS-MS/MS ^a^.

Ginsenosides	American Ginseng Root Residue (μg/g) ^b^	
	HPLC-DAD-ESI-MS	UPLC-HRMS-MS/MS
Re	9081.64 ± 35.94	7958.80 ± 139.61
Rg1	305.76 ± 16.77	934.56 ± 39.84
Rf	ND ^c^	4.71 ± 0.33 ^d^
Rb1	23,254.89 ± 210.27	21,256.18 ± 429.25
Rc	949.37 ± 27.69	1262.94 ± 58.00
Rb2	558.26 ± 1.62	583.68 ± 36.70
Rd	1300.39 ± 17.39	1935.39 ± 48.64
Rg3	ND	353.16 ± 10.98
CK	ND	ND
Total	35,450.31 ± 281.98	34,289.42 ± 101.61

^a^ The contents of ginsenosides in American ginseng root residue as determined by HPLC-DAD-ESI-MS and UPLC-HRMS-MS/MS are based on the respective quantitation method described in Section 3.4 and Section 3.6. ^b^ Mean of triplicate analyses ± standard deviation. ^c^ ND, not detected (below the limit of detection). ^d^ The value is lower than the limit of quantitation.

**Table 3:** The recovery data of ginsenosides by HPLC-DAD-ESI-MS ^a^.

Ginsenoside	Original (μg/g)	Spiked (μg/g)	Found (μg/g) ^b^	Recovery (%) ^c^	Mean ± SD (%) ^d^	RSD (%) ^e^
Rg1	485.76 ± 6.77	400	917.94 ± 37.95	108.04	103.83 ± 5.95	5.73
		200	685.01 ± 18.97	99.62		
Re	8291.64 ± 25.94	2000	10,526.76 ± 189.74	111.76	108.96 ± 3.95	3.63
		1000	9353.32 ± 94.87	106.17		
Rf	ND ^f^	120	124.34 ± 11.38	103.62	101.64 ± 2.80	2.75
		60	59.79 ± 5.69	99.66		
Rb1	23,194.89 ± 90.27	5000	28,082.08 ± 474.34	97.74	102.97 ± 7.40	7.18
		2500	25,900.01 ± 237.17	108.20		
Rc	889.37 ± 7.69	1000	1971.91 ± 94.87	108.25	102.50 ± 8.14	7.94
		500	1373.10 ± 47.43	96.75		
Rb2	248.26 ± 6.62	300	560.70 ± 28.46	104.15	104.52 ± 0.52	0.50
		150	405.59 ± 14.23	104.89		
Rd	1680.39 ± 27.39	500	2161.50 ± 47.43	96.22	103.37 ± 10.10	9.77
		250	1956.66 ± 23.72	110.51		
Rg3	ND	380	432.78 ± 36.05	113.89	114.32 ± 0.61	0.54
		190	218.04 ± 18.02	114.76		

^a^ The recovery data of ginsenosides by HPLC-DAD-ESI-MS are based on a method described in Section 3.5. ^b^ Mean ± standard deviation of five replicates. ^c^ Recovery = (amount found—original amount/amount spiked) × 100. ^d^ Mean ± standard deviation of recovery data from two spiked ginsenoside concentrations. ^e^ Relative standard deviation (%) = (standard deviation/mean) × 100. ^f^ ND, not detected (below the limit of detection).

**Table 4:** Repeatability and intermediate precision of ginsenosides by HPLC-DAD-ESI-MS ^a^.

Ginsenoside	Repeatability ^b,c^		Intermediate Precision ^c,d^	
	Mean ± SD (μg/g)	RSD (%) ^e^	Mean ± SD (μg/g)	RSD (%)
Rg1	485.76 ± 6.77	1.39	500.17 ± 8.37	1.68
Re	8291.64 ± 25.94	0.31	8143.70 ± 136.48	1.67
Rf	ND ^f^		ND	
Rb1	23,194.89 ± 90.27	0.39	22,988.69 ± 443.31	1.93
Rc	889.37 ± 7.69	0.86	927.87 ± 85.18	9.18
Rb2	248.26 ± 6.62	2.67	233.32 ± 13.85	5.94
Rd	1680.39 ± 27.39	1.63	1701.59 ± 39.11	2.30
Rg3	ND		ND	

^a^ The repeatability and intermediate precision data of ginsenosides by HPLC-DAD-ESI-MS are based on a method described in Section 3.5. ^b^ Mean ± standard deviation of five replicates on the same day. ^c^ Data obtained by spiking each ginsenoside at a concentration 10 times that of LOQ. ^d^ Mean ± standard deviation of fifteen replicates with five replicates per day for three consecutive days. ^e^ Relative standard deviation (%) = (standard deviation/mean) × 100. ^f^ ND, not detected (below the limit of detection).

**Table 5:** Contents (μg/g) of ginsenosides in American ginseng root residue as affected by different extraction solvents based on HPLC-DAD-ESI-MS analysis ^a,b,c^.

Ginsenosides	80% Methanol	30% Ethanol	50% Ethanol	80% Ethanol	95% Ethanol
Re	10,664.66 ± 188.85 ^A^	8632.90 ± 152.90 ^D^	10,358.99 ± 182.28 ^B^	9943.04 ± 175.42 ^C^	4904.90 ± 86.90 ^E^
Rg1	534.82 ± 8.68 ^A^	367.25 ± 6.95 ^C^	505.10 ± 8.72 ^B^	532.28 ± 8.71 ^A^	198.29 ± 3.34 ^D^
Rf	ND ^d^	ND	ND	ND	ND
Rb1	24,021.72 ± 458.36 ^A^	18,413.34 ± 338.49 ^C^	22,849.00 ± 420.86 ^B^	23,987.07 ± 433.37 ^A^	12,210.58 ± 224.34 ^D^
Rc	1371.15 ± 44.26 ^A^	1017.67 ± 32.74 ^C^	1315.60 ± 42.74 ^A^	1173.83 ± 37.19 ^B^	830.28 ± 26.30 ^D^
Rb2	ND	ND	ND	ND	ND
Rd	1158.93 ± 28.01 ^A^	780.22 ± 19.57 ^D^	990.65 ± 24.37 ^B^	831.64 ± 20.80 ^C^	525.74 ± 13.51 ^D^
Rg3	ND	ND	ND	ND	ND
total	37,751.28 ± 726.02 ^A^	29,211.38 ± 547.41 ^B^	36,019.34 ± 676.47 ^A^	36,467.86 ± 673.37 ^A^	18,669.79 ± 352.33 ^C^

^a^ The contents of ginsenosides in American ginseng root residue by HPLC-DAD-ESI-MS as affected by different extraction solvents are based on a method described in Section 3.3. ^b^ Mean of triplicate analyses ± standard deviation. ^c^ Symbols bearing different capital letters (A–E) in the same row are significantly different (*p* < 0.05). ^d^ ND = not detected (below the limit of detection).

**Table 6:** The precursor ion, product ion, collision energy, and radiofrequency (RF) lens conditions of 10 ginsenosides by UPLC-HRMS-MS/MS ^a^.

Peak No.	Ginsenoside	Retention Time (min)	*m*/*z* Value		Collision Energy (V)	RF Lens (V)
			Precursor Ion	Product Ion		
1	Rg1	2.45	800.54 [M-H]^−^	638.44 ^b^	22.6	125
				476.42 ^c^	31.1	125
2	Re	2.61	946.54 [M-H]^−^	638.44 ^b^	35.1	200
				476.54 ^c^	55.0	200
3	Rf	4.16	799.70 [M-H]^−^	475.38 ^b^	41.2	136
				637.44 ^c^	31.7	136
4	Rb1	4.27	1107.63 [M-H]^−^	220.98 ^b^	52.8	249
				945.48 ^c^	42.4	249
5	Rc	4.38	1077.60 [M-H]^−^	945.49 ^b^	41.0	195
				765.47 ^c^	47.3	195
6	Rb2	4.52	1077.60 [M-H]^−^	783.39 ^b^	47.9	213
				765.48 ^c^	44.9	213
7	Rd	4.80	945.70 [M-H]^−^	783.50 ^b^	37.3	205
				621.44 ^c^	40.6	205
8	Saikosaponin A (IS)	5.17	780.51 [M-H]^−^	618.45	33.4	144
9	Rg3	5.46	783.70 [M-H]^−^	621.43 ^b^	32.0	133
				459.37 ^c^	39.1	133
10	CK	5.76	621.54 [M-H]^−^	161.05 ^b,c^	18.5	103

^a^ The mass spectral identification data by UPLC-HRMS-MS/MS are based on a method described in Section 3.6. ^b^ product ion with high signal intensity used for quantitative analysis. ^c^ product ion with low signal intensity used for qualitative analysis.

**Table 7:** The recovery data of ginsenosides by UPLC-HRMS-MS/MS ^a^.

Ginsenoside	Original (μg/g)	Spiked (μg/g)	Found (μg/g) ^b^	Recovery (%) ^c^	Mean ± SD (%) ^d^	RSD (%) ^e^
Rg1	926.61 ± 33.32	900	1851.04 ± 49.19	102.19	97.91 ± 6.06	6.19
		450	1352.66 ± 56.51	93.62		
Re	7977.10 ± 137.02	2000	9641.92 ± 237.30	83.24	87.66 ± 6.25	7.12
		1000	8897.83 ± 189.69	92.07		
Rf	4.49 ± 0.40	5	9.26 ± 0.35	93.88	86.82 ± 9.99	11.51
		2.5	6.56 ± 0.63	79.75		
Rb1	21,339.42 ± 334.71	5000	25,444.87 ± 439.81	82.11	99.15 ± 24.09	24.30
		2500	24,243.99 ± 687.65	116.18		
Rc	1260.05 ± 46.91	1000	2281.50 ± 86.17	103.06	100.83 ± 3.15	3.13
		500	1743.92 ± 55.83	98.60		
Rb2	582.04 ± 34.08	500	1052.85 ± 49.45	94.16	97.21 ± 4.31	4.43
		250	832.68 ± 41.66	100.26		
Rd	1931.10 ± 44.44	400	2401.81 ± 46.37	95.28	100.82 ± 7.83	7.76
		200	2181.64 ± 33.53	106.35		
Rg3	315.34 ± 8.69	200	491.27 ± 13.79	98.17	93.88 ± 6.06	6.45
		100	416.23 ± 17.01	89.60		

^a^ The recovery data of ginsenosides by UPLC-HRMS-MS/MS are based on a method described in Section 3.7. ^b^ Mean ± standard deviation of five replicates. ^c^ Recovery = (amount found—original amount/amount spiked) × 100. ^d^ Mean ± standard deviation of recovery data from two spiked ginsenoside concentrations. ^e^ Relative standard deviation (%) = (standard deviation/mean) × 100.

**Table 8:** Repeatability and intermediate precision of ginsenosides by UPLC-HRMS-MS/MS ^a^.

Ginsenoside	Repeatability ^b,c^		Intermediate Precision ^c,d^	
	Mean ± SD (μg/g)	RSD (%) ^e^	Mean ± SD (μg/g)	RSD (%)
Rg1	994.56 ± 59.84	6.02	994.62 ± 65.00	6.53
Re	8828.80 ± 229.61	2.60	8538.95 ± 464.67	5.44
Rf	4.71 ± 0.33	7.08	4.51 ± 0.38	8.31
Rb1	20,096.18 ± 289.25	1.44	20,446.33 ± 1113.67	5.45
Rc	1092.94 ± 38.00	3.48	1147.84 ± 86.88	7.57
Rb2	543.68 ± 26.70	4.91	550.35 ± 31.66	5.75
Rd	1985.39 ± 88.64	2.30	1939.26 ± 72.89	3.76
Rg3	343.16 ± 10.98	3.20	340.02 ± 13.36	3.93

^a^ The repeatability and intermediate precision data of ginsenosides by UPLC-HRMS-MS/MS are based on a method described in Section 3.7. ^b^ Mean ± standard deviation of five replicates on the same day. ^c^ Data obtained by spiking each ginsenoside at a concentration 10 times that of LOQ. ^d^ Mean ± standard deviation of fifteen replicates with five replicates per day for three consecutive days. ^e^ Relative standard deviation (%) = (standard deviation/mean) × 100.

## Data Availability

The data presented in this study are available in this article.
